# Draft Genome Sequence of Heavy Metal-Resistant Aeromonas veronii CTe-01, Isolated from a Peruvian Wastewater Treatment Plant

**DOI:** 10.1128/MRA.01147-19

**Published:** 2019-12-05

**Authors:** Luis Tataje-Lavanda, Phillip Ormeño-Vásquez, Rosa Altamirano-Díaz, Lucy Espinoza-Salazar, Mirko Zimic, Manolo Fernández-Sánchez, Manolo Fernández-Díaz, Claudio C. Vásquez, Juan C. Tantaleán

**Affiliations:** a Laboratorios de Investigación y Desarrollo, FARVET, Chincha Alta, Ica, Peru; b Laboratorio de Bioinformática, Biología Molecular y Desarrollos Tecnológicos, Laboratorios de Investigación y Desarrollo, Facultad de Ciencias, Universidad Peruana Cayetano Heredia, Lima, Peru; c Laboratorio de Biología Molecular y Biotecnología, Facultad de Ciencias Biológicas, Universidad Nacional San Luis Gonzaga, Ica, Peru; d Laboratorio de Microbiología Molecular, Facultad de Química y Biología, Universidad de Santiago de Chile, Santiago, Chile; Georgia Institute of Technology

## Abstract

Here, we report a draft genome sequence of Aeromonas veronii strain CTe-01 (4.5 Mb), a hemolytic, heavy metal-resistant bacterium isolated from a wastewater treatment plant located at Cachiche, Ica, Peru. These characteristics could be used for bioremediation of contaminated environments.

## ANNOUNCEMENT

Aeromonas veronii causes infections and death in fish (1, 2) and other freshwater animals (3). It is an emerging pathogen involved in enteric (4) and extraintestinal infections in humans (5–8), and its pathogenicity is usually multifactorial (6). Additionally, it is routinely oxacillin, ampicillin, tetracycline, streptomycin, chloramphenicol, and erythromycin resistant (1, 9). It is typically a Gram-negative, lactose-negative, and oxidase-positive bacterium that has been isolated from soils, food, animals, and different aquatic sources (10, 11). Studies of heavy metal resistance genes in *A. veronii* are limited, so the study of its genome is important because, like other *Aeromonas* species, it harbors heavy metal resistance genes, including those for mercury, cobalt, zinc, cadmium, and chromium resistance, among others (12). Frequently, *A. veronii* harbors mobile elements, such as plasmids, insertion sequences (IS), transposons, genes associated with integrons, and bacteriophages (12–14), which participate in horizontal transfer and diffusion of heavy metal resistance and antibiotic resistance to other environmental bacteria (15–17).

*A. veronii* CTe-01 was isolated from a wastewater sample at Cachiche, Ica, Peru (14°07′06.00ʺS, 75°43′34.89ʺW; 391 m above sea level [masl]) using Luria Bertani (LB) medium supplemented with potassium tellurite (75 μg/ml). It is a hemolytic bacterium which grows in the presence of HgCl_2_ (10 μg/ml). For its identification, biochemical characteristics were evaluated using an automatic Vitek 2 compact system with Gram-negative (GN) bacterial identification (ID) cards (bioMérieux), and the 16S rRNA gene was sequenced by Macrogen, Inc. (USA) (GenBank accession number MK876839.1).

Pure cultures were grown in Luria Bertani broth containing 100 μg/ml ampicillin and incubated at 37°C for 12 h. Genomic DNA was extracted using a DNeasy blood and tissue kit (Qiagen, Germany) and sequenced at Macrogen, Inc. (South Korea) using a HiSeq 2000 platform with 101-bp paired-end reads (TruSeq DNA PCR-free library), producing 2,145,830,850 bp with a total of 21,245,850 reads. Unless otherwise stated, default parameters were used for all software. Reads were checked for quality using FastQC version 0.11.5 (Babraham Bioinformatics, Cambridge, UK) and assembled *de novo* using SPAdes version 3.11 (with the “-careful” and “-plasmid” settings) (18) without trimming the reads to avoid losing information; contigs were reordered with Mauve version 2015-02-26 (19). After removal of small contigs (<200 bp) and contaminants, 272 contigs were obtained (total length, 4,641,822 bp; *N*_50_, 79,963 bp; *L*_50_, 22; GC content, 58.64%; genome coverage, ∼383×) (QUAST Web interface [20]). Annotation was carried out using the NCBI Prokaryotic Genome Automatic Annotation Pipeline version 4.8 (21) and predicted 4,332 genes, 4,283 coding DNA sequences (CDS), 49 RNA genes (4 rRNAs, 40 tRNAs, 5 noncoding RNAs [ncRNAs]), 162 pseudogenes, and 1 CRISPR array. ResFinder version 3.2 analyses (DTU, Denmark) (22) identified 34 beta-lactam resistance genes and 1 colistin resistance gene (see Table I at https://doi.org/10.6084/m9.figshare.9828173).

Analyses of metal resistance were performed using BLASTX with the BacMet2 experimentally confirmed resistance gene database version 2.0 (http://bacmet.biomedicine.gu.se/download/BacMet2_EXP_database.fasta) (23). A total of 195 genes were found to be associated with the bacterial chromosome, 25 genes were associated with plasmids, and 5 genes were present in both genetic elements. Resistance genes for Cu (*n* = 30), Zn (*n* = 23), Co (*n* = 19), Ni (*n* = 17), As (*n* = 13), Cd (*n* = 12), Fe (*n* = 11), Te (*n* = 10), and others (Sb, Ag, Cr, Hg, Mn, W, Mg, Se, Au, Mo, Al, V, Ga, and Pb) were identified (see Table II at https://doi.org/10.6084/m9.figshare.9828173).

### Data availability.

The genome sequence of CTe-01 has been deposited at the GenBank database under the accession number VATZ00000000. The raw data are available at the SRA under run number SRR9052997.

## References

[1] de Jagoda SS, Wijewardana TG, Arulkanthan A, Igarashi Y, Tan E, Kinoshita S, Watabe S, Asakawa S 2014 Characterization and antimicrobial susceptibility of motile aeromonads isolated from freshwater ornamental fish showing signs of septicaemia. Dis Aquat Organ 109:127–137. doi:10.3354/dao02733. 24991740

[2] Walczak N, Puk K, Guz L 2017 Bacterial flora associated with diseased freshwater ornamental fish. J Vet Res 61:445–449. doi:10.1515/jvetres-2017-0070. 29978108 PMC5937343

[3] Liu ZG, Zheng AF, Chen MM, Lian YX, Zhang XK, Zhang SZ, Yu D, Li JK 2018 Isolation and identification of pathogenic Aeromonas veronii from a dead Yangtze finless porpoise. Dis Aquat Organ 132:13–22. doi:10.3354/dao03288. 30530927

[4] da Silva LCA, Leal-Balbino TC, de Melo BST, Mendes-Marques CL, Rezende AM, de Almeida AMP, Leal NC 2017 Genetic diversity and virulence potential of clinical and environmental Aeromonas spp. isolates from a diarrhea outbreak. BMC Microbiol 17:179. doi:10.1186/s12866-017-1089-0. 28821241 PMC5563053

[5] Janda JM, Abbott SL 2010 The genus Aeromonas: taxonomy, pathogenicity, and infection. Clin Microbiol Rev 23:35–73. doi:10.1128/CMR.00039-09. 20065325 PMC2806660

[6] Igbinosa IH, Igumbor EU, Aghdasi F, Tom M, Okoh AI 2012 Emerging Aeromonas species infections and their significance in public health. ScientificWorldJournal 2012:625023. doi:10.1100/2012/625023. 22701365 PMC3373137

[7] Monti M, Torri A, Amadori E, Rossi A, Bartolini G, Casadei C, Frassineti GL 2019 Aeromonas veronii biovar veronii and sepsis—infrequent complication of biliary drainage placement: a case report. World J Clin Cases 7:759–764. doi:10.12998/wjcc.v7.i6.759. 30968041 PMC6448070

[8] Zhou Y, Yu L, Nan Z, Zhang P, Kan B, Yan D, Su J 2019 Taxonomy, virulence genes and antimicrobial resistance of Aeromonas isolated from extra-intestinal and intestinal infections. BMC Infect Dis 19:158. doi:10.1186/s12879-019-3766-0. 30764764 PMC6376669

[9] Vincent AT, Trudel MV, Paquet VE, Boyle B, Tanaka KH, Dallaire-Dufresne S, Daher RK, Frenette M, Derome N, Charette SJ 2014 Detection of variants of the pRAS3, pAB5S9, and pSN254 plasmids in Aeromonas salmonicida subsp. salmonicida: multidrug resistance, interspecies exchanges, and plasmid reshaping. Antimicrob Agents Chemother 58:7367–7374. doi:10.1128/AAC.03730-14. 25267667 PMC4249516

[10] Skwor T, Shinko J, Augustyniak A, Gee C, Andraso G 2014 Aeromonas hydrophila and Aeromonas veronii predominate among potentially pathogenic ciprofloxacin- and tetracycline-resistant Aeromonas isolates from Lake Erie. Appl Environ Microbiol 80:841–848. doi:10.1128/AEM.03645-13. 24242249 PMC3911203

[11] Neupane S, Modry D, Pafčo B, Zurek L 2019 Bacterial community of the digestive tract of the European medicinal leech (Hirudo verbana) from the Danube River. Microb Ecol 77:1082–1090. doi:10.1007/s00248-019-01349-z. 30806729

[12] Abdelhamed H, Lawrence ML, Waldbieser G 2019 Complete genome sequence data of multidrug-resistant Aeromonas veronii strain MS-18–37. Data Brief 23:103689. doi:10.1016/j.dib.2019.01.037. 30788401 PMC6369315

[13] Piotrowska M, Popowska M 2015 Insight into the mobilome of Aeromonas strains. Front Microbiol 6:494. doi:10.3389/fmicb.2015.00494. 26074893 PMC4444841

[14] Roh HJ, Kim B-S, Kim A, Kim NE, Lee Y, Chun W-K, Ho TD, Kim D-H 2019 Whole-genome analysis of multi-drug-resistant Aeromonas veronii isolated from diseased discus (Symphysodon discus) imported to Korea. J Fish Dis 42:147–153. doi:10.1111/jfd.12908. 30350465

[15] Bennett PM 2008 Plasmid encoded antibiotic resistance: acquisition and transfer of antibiotic resistance genes in bacteria. Br J Pharmacol 153:S347–S357. doi:10.1038/sj.bjp.0707607. 18193080 PMC2268074

[16] Aminov RI 2011 Horizontal gene exchange in environmental microbiota. Front Microbiol 2:158. doi:10.3389/fmicb.2011.00158. 21845185 PMC3145257

[17] Rodríguez-Blanco A, Lemos ML, Osorio CR 2012 Integrating conjugative elements as vectors of antibiotic, mercury, and quaternary ammonium compound resistance in marine aquaculture environments. Antimicrob Agents Chemother 56:2619–2626. doi:10.1128/AAC.05997-11. 22314526 PMC3346659

[18] Bankevich A, Nurk S, Antipov D, Gurevich AA, Dvorkin M, Kulikov AS, Lesin VM, Nikolenko SI, Pham S, Prjibelski AD, Pyshkin AV, Sirotkin AV, Vyahhi N, Tesler G, Alekseyev MA, Pevzner PA 2012 SPAdes: a new genome assembly algorithm and its applications to single-cell sequencing. J Comput Biol 19:455–477. doi:10.1089/cmb.2012.0021. 22506599 PMC3342519

[19] Darling ACE, Mau B, Blattner FR, Perna NT 2004 Mauve: multiple alignment of conserved genomic sequence with rearrangements. Genome Res 14:1394–1403. doi:10.1101/gr.2289704. 15231754 PMC442156

[20] Gurevich A, Saveliev V, Vyahhi N, Tesler G 2013 QUAST: quality assessment tool for genome assemblies. Bioinformatics 29:1072–1075. doi:10.1093/bioinformatics/btt086. 23422339 PMC3624806

[21] Tatusova T, DiCuccio M, Badretdin A, Chetvernin V, Nawrocki EP, Zaslavsky L, Lomsadze A, Pruitt KD, Borodovsky M, Ostell J 2016 NCBI Prokaryotic Genome Annotation Pipeline. Nucleic Acids Res 44:6614–6624. doi:10.1093/nar/gkw569. 27342282 PMC5001611

[22] Zankari E, Hasman H, Cosentino S, Vestergaard M, Rasmussen S, Lund O, Aarestrup FM, Larsen MV 2012 Identification of acquired antimicrobial resistance genes. J Antimicrob Chemother 67:2640–2644. doi:10.1093/jac/dks261. 22782487 PMC3468078

[23] Pal C, Bengtsson-Palme J, Rensing C, Kristiansson E, Larsson D 2014 BacMet: antibacterial biocide and metal resistance genes database. Nucleic Acids Res 42:D737–D43. doi:10.1093/nar/gkt1252. 24304895 PMC3965030

